# Statewide Prevalence of Smoke-Free and Vape-Free Homes, by Tobacco Product Use, Minnesota, 2018

**DOI:** 10.5888/pcd17.200133

**Published:** 2020-11-12

**Authors:** Sharrilyn Helgertz, Ann St. Claire, John Kingsbury

**Affiliations:** 1 Minnesota Department of Health, St Paul, Minnesota; 2 ClearWay Minnesota, Minneapolis, Minnesota

## Abstract

**Introduction:**

Securing clean indoor air laws is a major tobacco control accomplishment of the past 15 years. The public quickly adopted and supported such policies both in public and private spaces. Clean indoor air is now threatened by the emergence of e-cigarettes. E-cigarette aerosol contains nicotine, heavy metals, and carcinogens, and the long-term effect of secondhand exposure is unknown. Surveillance is necessary to track voluntary rules on smoking and vaping in the home.

**Methods:**

The Minnesota Adult Tobacco Survey (MATS) is a series of cross-sectional, random-digit–dial telephone surveys on smoking, vaping, and other tobacco-related behaviors, attitudes, and beliefs among Minnesota adults. MATS measured voluntary smoke-free rules in the home in 2014 (N = 9,304) and measured both smoke-free and vape-free home rules in 2018 (N = 6,055).

**Results:**

The prevalence of smoke-free home rules among Minnesota adults in 2018 was 91.5% (95% CI, 90.5%–92.5%), up slightly from 89.3% (95% CI, 88.4%–90.2%) in 2014. In comparison, 84.0% (95% CI, 82.7%–85.3%) reported vape-free home rules. Although 70.0% (95% CI, 66.0%–73.0%) of smokers in 2018 reported smoke-free home rules, only 23.3% (95% CI, 15.0%–31.6%) of e-cigarette users reported vape-free home rules. Living with children younger than 18 years significantly increased the odds of having smoke-free and vape-free home rules.

**Conclusion:**

Although widespread adoption of voluntary smoke-free and vape-free home rules demonstrates a positive change in social norms, most e-cigarette users allow vaping in their homes, including those who live with children younger than 18. Tracking voluntary smoke-free and vape-free home rules and efforts to encourage them are important to improve the public’s health.

**Summary**

**What is already known on this topic?**

Adults are increasingly making their homes smoke-free and protecting children and other nonsmokers from dangerous secondhand smoke. E-cigarettes pose a new threat to clean indoor air, but few studies have examined whether homes are vape-free.

**What is added by this report?**

This study provides the first statewide estimate of vape-free rules and shows that most adults who use e-cigarettes, including those who live with children, allow vaping in their homes and are potentially exposing others to secondhand aerosol.

**What are the implications for public health practice?**

Clean indoor air efforts should promote smoke-free and vape-free homes.

## Introduction

Securing clean indoor air laws is a major tobacco control accomplishment of the past 15 years. Still, secondhand tobacco smoke poses a serious health threat to nonsmokers (1). Strong clean indoor air laws have reduced exposure to secondhand smoke in public places (2) and encouraged adoption of smoke-free rules in private spaces (3,4), but the home remains the primary source of secondhand exposure for children and a major source of exposure for nonsmoking adults (1). Although the number of households with smoke-free rules has increased over time (5), adoption of smoke-free home rules is uneven. Households with children are more likely to have rules against smoking in the home; households with adults who are older, have lower income, have less education, or smoke, are less likely (6,7).

Emerging in 2007, e-cigarettes have been marketed as a healthier alternative to combustible cigarettes, and some advertisements promoted their use where smoking is not allowed (8). E-cigarette aerosol contains nicotine, heavy metals, carcinogens, ultra-fine particulate matter exceeding background levels, and metals, such as nickel and chromium, that exceed levels associated with conventional smoking (9). Although the long-term risks of secondhand aerosol exposure are unknown, studies show that e-cigarette use contaminates the air under controlled (10) and real-world conditions (11), extending the potential health risks beyond the user (12).

Several state and local governments have added e-cigarettes to their smoke-free laws. However, misleading industry marketing, acceptance of e-cigarettes by some public health advocates as a harm-reduction strategy for smokers, and slow regulation by the US Food and Drug Administration have contributed to the public’s perception that e-cigarette aerosol is low risk or even harmless (13). Adults who prohibit smoking conventional cigarettes in their homes may allow vaping and unwittingly expose friends and family to potential health risks. Our study uses data from the Minnesota Adult Tobacco Survey (MATS) to describe the first statewide prevalence of vape-free home rules and examine whether e-cigarette use and children in the home predict adoption of vape-free home rules.

## Methods

Data came from the 2014 and 2018 administrations of MATS. MATS is a series of cross-sectional, random-digit–dial landline and cell phone surveys of civilian, noninstitutionalized adults aged 18 or older living in Minnesota. MATS data were collected in 1999, 2003, 2007, 2010, 2014, and 2018. The MATS 2014 final sample included 9,304 respondents; the response rate was 25.2% for the landline survey and 18.2% for the cell phone survey. In 2018, MATS included 6,055 participants and yielded American Association for Public Opinion Research (AAPOR) response rates of 17.5% for the landline survey and 13.4% for the cell phone survey. The same screening, sampling, and refusal conversion protocols were used in both survey years. Weighting was applied to create unbiased population estimates based on the probability of selection resulting from the sampling plan. Weights were calibrated based on sex, race/ethnicity, location, and education totals from the US Census Bureau’s American Community Survey (14). Methodologic details are available at www.clearwaymn.org/MATS. MATS was approved by the Minnesota Department of Health’s institutional review board.

### Measures

MATS measured voluntary smoke-free rules in the home in 2014 and 2018 and vape-free rules in 2018. Smoke-free rules were measured by the question, “Which statement best describes rules about smoking inside your home (excluding porches and garages)?” Vape-free rules were measured similarly: “Which statement best describes the rules about using e-cigarettes or vaping devices inside your home (excluding porches and garages)?” Responses included not allowed anywhere, allowed in some places or at some times, or allowed anywhere. Only respondents who indicated that smoking or vaping was not allowed anywhere were considered to have rules against that activity.

Covariates were age (4 categories), sex (male or female), race/ethnicity (5 mutually exclusive categories), education (4 categories), annual household income (4 categories), marital status (married or not), current smoker of cigarettes, cigars, or pipe (yes or no), current e-cigarette user (yes or no), and lives with a child younger than 18 years (yes or no). Respondents who reported currently smoking cigarettes, cigars, or pipe every day or some days and met minimum lifetime-use thresholds (100 cigarettes, 20 times, or 20 times, respectively) were categorized as current smokers. Similarly, respondents who reported currently using e-cigarettes every day or some days were categorized as current e-cigarette users; however, no minimum lifetime use was required.

### Analysis

We performed all analyses using SPSS Statistics version 24 (IBM Corporation) for complex samples. We used pairwise deletion to maximize available data. We used the Pearson χ^2^ to assess the change from 2014 to 2018 in the percentage of Minnesota adults with smoke-free rules and assess the bivariate association between smoke-free and vape-free rules and respondent characteristics. We used logistic regression to assess the unique association between respondent characteristics and smoke-free rules and vape-free rules in 2018. We entered the entire set of potential predictors simultaneously, testing for main effects only. Ordinal variables were dummy coded. We used odds ratios (ORs) and 95% CIs to estimate the likelihood of adults reporting smoke-free or vape-free rules.

## Results

The prevalence of smoke-free home rules among Minnesota adults in 2018 was 91.5% (95% CI, 90.5%–92.5%), up slightly from 89.3% (95% CI, 88.4%–90.2%) in 2014 (χ_1_^2^ = 21.8; *P* < .001). Current smokers were less likely than nonsmokers to report having smoke-free rules (70.0%; 95% CI, 66.0%–74.0% vs 96.2%; 95% CI, 95.6%–96.8%). The percentage of smokers with smoke-free rules in 2018 was not significantly different from the percentage observed in 2014 (65.5%; 95% CI, 63.0%–68.02%).

In the bivariate analysis, having smoke-free rules was significantly associated with age, sex, race/ethnicity, education, annual household income, marital status, smoking status, e-cigarette use, and children younger than 18 years in the home (Table 1).

**Table 1:** Prevalence of Smoke-Free Rules and Vape-Free Rules Among Minnesota Adults, Minnesota Adult Tobacco Survey, 2018

Characteristic	Unweighted No. (%)^a^	Smoke-Free Home, % (95% CI)^b^	Vape-Free Home, % (95% CI)^b^
**Overall**	5,538 (100.0)	91.5 (90.5–92.5)	84.0 (82.7–85.3)
**Age, y**			
18–24	421 (7.6)	91.1 (88.5–93.7)	68.7 (63.7–73.7)
25–44	1,318 (23.8)	93.4 (91.9–94.9)	83.1 (80.9–85.3)
45–64	1,950 (35.2)	89.4 (87.6–91.2)	85.4 (83.4–87.4)
≥65	1,849 (33.4)	92.3 (90.8–93.8)	93.2 (91.9–94.5)
**Sex**			
Male	2,569 (46.4)	90.2 (88.8–91.6)	81.6 (79.7–83.5)
Female	2,969 (53.6)	92.8 (91.5–94.1)	86.3 (84.7–87.9)
**Race/ethnicity**			
Non-Hispanic White	4,550 (82.2)	92.0 (91.0–93.0)	84.4 (83.0–85.8)
Hispanic	271 (4.9)	94.5 (91.6–97.4)	85.0 (79.4–90.6)
Multi/other	254 (4.6)	79.9 (70.6–89.2)	64.8 (55.3–74.3)
Non-Hispanic Black	239 (4.3)	84.9 (79.6–90.2)	83.8 (78.5–89.1)
Asian	153 (2.8)	94.5 (91.7–97.3)	87.7 (82.7–92.7)
**Education**			
<High school graduate	182 (3.3)	82.5 (76.4–88.6)	71.6 (64.0–79.2)
High school graduate/GED	1,180 (21.3)	87.3 (85.2–89.4)	82.8 (80.4–85.2)
Some college or technical school	1,797 (32.4)	91.3 (89.8–92.8)	80.6 (78.2–83.0)
≥College graduate	2,346 (42.4)	97.0 (96.3–97.7)	91.3 (90.0–92.6)
**Annual household income, $**			
≤35,000	1,142 (20.6)	83.9 (81.0–86.8)	74.5 (71.0–78.0)
35,001–50,000	590 (10.7)	89.7 (87.1–92.3)	82.9 (79.3–86.5)
50,001–75,000	878 (15.9)	92.3 (90.1–94.5)	84.8 (81.8–87.8)
≥75,001	2,247 (40.6)	95.4 (94.3–96.5)	88.1 (86.4–89.8)
**Marital status**			
Married	2,896 (52.3)	94.9 (93.9–95.9)	90.6 (89.3–91.9)
Not married	2,617 (47.3)	87.5 (85.8–89.2)	76.2 (73.9–78.5)
**Current smoker of cigarettes, cigars, or pipe **			
Yes	765 (13.8)	70.0 (66.0–74.0)	55.8 (51.3–60.3)
No	4,752 (85.8)	96.2 (95.6–96.8)	90.2 (89.0–91.4)
**Current e-cigarette user**			
Yes, every/some days	178 (3.2)	80.1 (73.9–86.3)	23.3 (15.0–31.6)
No, not at all	5,359 (96.8)	92.1 (91.2–93.0)	87.0 (85.8–88.2)
**Children in the home**			
Yes	1,486 (26.8)	95.2 (94.0–96.4)	87.6 (85.7–89.5)
No	4,048 (73.1)	89.4 (88.0–90.8)	81.9 (80.3–83.5)

Abbreviation: GED, general equivalency diploma.

a Percentages are based on the value in the column heading; some percentages may not sum to 100 because of missing data (respondents declined to answer question or responded with “don’t know”).

b All Pearson χ^2^ tests of independence between smoke-free and vape-free home rules were significant at *P* <.01.

More than 4 in 5 adults (84.0%; 95% CI, 82.7%–85.3%) reported vape-free home rules in 2018. Having vape-free rules was significantly associated with all respondent characteristics assessed (Table 1). The prevalence of vape-free home rules was considerably lower among adults aged 18 to 24 (68.7%; 95% CI, 63.7%–73.7%) than among adults 65 or older (93.2%; 95% CI, 91.9%–94.5%). Only 23.3% (95% CI, 15.0%–31.6%) of current e-cigarette users reported vape-free homes. The percentage of adults who reported vape-free home rules was higher among respondents living with children younger than 18 years (87.6%; 95% CI, 85.7%–89.5%) than among those not living with children younger than 18 years (81.9%; 95% CI, 80.3%–83.5%).

**Association of respondent characteristics with smoke-free rules in the multivariate analysis.** When we controlled for other variables in the model, respondent sex, annual household income, and e-cigarette use status were no longer associated with smoke-free home rules (Table 2). Adults who were aged 45 to 64 (OR = 0.58; 95% CI, 0.34–0.99) or Black (OR = 0.46; 95% CI, 0.24–0.88) were less likely to have smoke-free rules than adults aged 18 to 24 or White, respectively. Adults who had a college degree or higher (OR = 1.99; 95% CI, 1.02–3.90) or who were married (OR = 1.66; 95% CI, 1.16–2.39) were more likely to have smoke-free rules than adults who had less education or were not married, respectively. Nonsmokers were more likely to have smoke-free rules than smokers (OR = 9.12; 95% CI, 6.52–12.76). Adults who lived with children younger than 18 years were more likely to report smoke-free rules than those not living with children younger than 18 years (OR = 2.13; 95% CI, 1.37–3.32). The percentage of smokers who reported smoke-free home rules was greater among those who lived with children (82.0%; 95% CI, 76.8%–87.2%) than among those who did not (63.6%; 95% CI, 58.5%–68.7%).

**Table 2:** Adjusted and Weighted Odds of Smoke-Free and Vape-Free Rules Among Minnesota Adults, Minnesota Adult Tobacco Survey, 2018

Characteristics	Have Smoke-Free Rules, Odds Ratio (95% CI)	Have Vape-Free Rules, Odds Ratio (95% CI)
**Age, y**		
18–24	1 [Reference]	1 [Reference]
25–44	1.16 (0.66–2.04)	1.53 (1.03–2.26)^a^
45–64	0.58 (0.34–0.99)^a^	1.80 (1.21–2.67)^a^
≥65	0.62 (0.35–1.10)	3.54 (2.23–5.60)^a^
**Sex**		
Male	1 [Reference]	1 [Reference]
Female	1.23 (0.90–1.69)	1.17 (0.92–1.50)
**Race/ethnicity**		
White	1 [Reference]	1 [Reference]
Hispanic	1.27 (0.52–3.09)	1.23 (0.65–2.31)
Multi/other	0.55 (0.23–1.29)	0.57 (0.30–1.05)
Black	0.46 (0.24–0.88)^a^	1.16 (0.68–1.99)
Asian	0.61 (0.24–1.51)	1.11 (0.52–2.38)
**Education**		
<High school graduate	1 [Reference]	1 [Reference]
High school graduate/GED	0.88 (0.48–1.62)	1.18 (0.67–2.09)
Some college or technical school	1.14 (0.61–2.12)	0.86 (0.49–1.50)
≥College graduate	1.99 (1.02–3.90)^a^	1.30 (0.73–2.33)
**Annual household income, $**		
≤35,000	1 [Reference]	1 [Reference]
35,001–50,000	1.15 (0.72–1.84)	1.44 (0.98–2.13)
50,001–75,000	1.17 (0.72–1.90)	1.40 (0.95–2.07)
≥75,001	1.58 (0.99–2.52)	1.60 (1.13–2.26)^a^
**Marital status**		
Married	1.66 (1.16–2.39)^a^	1.31 (0.99–1.74)
Not married	1 [Reference]	1 [Reference]
**Current smoker of cigarettes, cigars, or pipe**		
Yes	1 [Reference]	1 [Reference]
No	9.12 (6.52–12.76)^a^	4.73 (3.61–6.20)
**Current e-cigarette user**		
Yes, every/some days	1 [Reference]	1 [Reference]
No, not at all	1.41 (0.78–2.56)	13.76 (8.11–23.36)^a^
**Children in the home**		
Yes	2.13 (1.37–3.32)^a^	1.87 (1.37–2.54)^a^
No	1 [Reference]	1 [Reference]

a Significant at *P* < .05.

**Association of respondent characteristics with vape-free rules in the multivariate analysis.** When we controlled for other variables in the model, sex, race/ethnicity, education, and marital status were no longer associated with vape-free home rules (Table 2). Adults who were older than 25 or had household incomes higher than $75,000 were more likely than adults younger than 25 or lower-income adults to report having vape-free rules. Nonsmokers were more likely than smokers to report vape-free rules (OR = 4.73; 95% CI, 3.61–6.20). Adults who lived with children younger than 18 years were more likely than those not living with children younger than 18 years to report vape-free rules (OR = 1.87; 95% CI, 1.37–2.54). Non–e-cigarette users were more likely than e-cigarette users to report vape-free rules (OR = 13.76, 95% CI, 8.11–23.36). Less than one-third of adults who used e-cigarettes and lived with children (29.4%; 95% CI, 13.1%–45.7%) reported vape-free home rules compared to 19.7% (95% CI, 10.2%–29.2%) of adults who vaped and did not live with children.

## Discussion

The prevalence of smoke-free home rules among Minnesota adults in 2018 was 91.5%, up slightly from 89.3% in 2014, and a substantial increase from 64.5% in 1999 (15). As smoke-free home rules approach universal adoption, persistent disparities in the prevalence of smoke-free home rules are beginning to disappear. In 2018, although older and lower-income adults continued to be less likely to report smoke-free rules than younger or higher-income adults, men and women and adults with varying levels of education were equally likely to report rules against smoking in the home.

However, the emergence of e-cigarettes and the potential for exposure to secondhand aerosol present a new threat to clean indoor air. Our study presents the first statewide estimate of vape-free home rules. In Minnesota, more than 4 in 5 adults (84.0%) have rules against vaping in the home, but as expected, this percentage was lower than the percentage of adults that have smoke-free home rules (91.5%). Although the prevalence of vape-free home rules is moderately high overall, our findings underscore the importance of targeting messages to segments of the population that do not have vape-free home rules. The discrepancy between smoke-free and vape-free rules may be due to the belief by many that electronic cigarettes are less harmful than conventional cigarettes (16). This belief may inappropriately trivialize the risk of secondhand aerosol exposure. A 2017 survey testing public awareness of aerosol constituents showed that 58% of US adults were unaware that e-cigarette aerosol contains more than water vapor; Black adults and smokers were least likely to have correct knowledge, and correct knowledge was associated with higher perceived harmfulness of secondhand exposure to aerosol (17). Education campaigns warning the public of the potential harms of secondhand aerosol exposure are needed.

Disparities in vape-free rules show marked similarity to historical disparities in smoke-free rules (6,7,15). Our study suggests that having vape-free rules, similar to having smoke-free rules, is largely a function of using the products oneself and having someone in the home to protect from exposure. Adults who use e-cigarettes are less likely to have vape-free rules, whereas adults who live with children younger than 18 years are more likely to prohibit vaping in the home. We observed a stark contrast among those who use these products between allowing smoking and vaping in homes where children are present. Among adults who live with children, 82.0% of smokers do not smoke in their homes, whereas only 29.4% of e-cigarette users do not vape in their homes.

Given the popularity of vaping and low prevalence of vape-free home rules among e-cigarette users, the advocacy message should now change from encouraging smoke-free rules to encouraging *clean air* rules in the home — no smoking or vaping in the home —especially in homes where children live. Smoke-free home rules have played a unique role in discouraging smoking (and increasing quitting [18]) by reducing social acceptance of the behavior and making smoking less convenient, and this phenomenon can likely be extended to vaping. As smoke-free policies in public places are amended to include e-cigarette use and public awareness of the potential harms of secondhand aerosol exposure increases, we anticipate voluntary vape-free rules will also increase. Researchers and public health practitioners can learn from the success of smoke-free efforts and build on existing practice to expedite additional public and private policies and communication campaigns to reduce the harm of secondhand smoke and aerosol exposure. Dedicating resources to successfully educate and promote vape-free home rules will protect nonusers from aerosol exposure and potentially contribute to youth vaping prevention. To inform intervention efforts, it would be helpful if future research explored the characteristics of people who allow vaping in their homes, especially those who allow vaping but not smoking, because this group may be particularly responsive to education on the risks of secondhand aerosol.

Our study has limitations. MATS did not assess the perceived harmfulness of secondhand aerosol or whether others in the respondent’s household vape, so we could not include these variables — which are likely to be associated with vape-free rules — in our analysis. Our study relies on self-report, and self-reported data are subject to some degree of social desirability and recall biases. Our data are from a state with strong antismoking norms and policies, so the findings may not generalize nationally or to other states.

Although widespread adoption of voluntary smoke-free and vape-free home rules demonstrates positive social norm change, most e-cigarette users in our study allowed vaping in their homes, including those who live with children younger than 18 years. Tracking voluntary smoke-free and vape-free home rules is an important component of tobacco control and demonstrates where resources should be directed to improve the public’s health.
